# Metabolic control of NETosis: dunnione suppresses inflammatory responses through NQO1–dependent redox modulation

**DOI:** 10.3389/fimmu.2026.1973758

**Published:** 2026-10-22

**Authors:** Meng-Yu Zhu, Seung-Hoon Lee, Gi-Su Oh, Jong-Suk Kim, Jae Ho Seo, Hong-Seob So

**Affiliations:** 1 Biomedical Science, Wonkwang University School of Medicine, Iksan, Republic of Korea; 2 Center for Metabolic Function Regulation (CMFR) and Department of Microbiology, Wonkwang University School of Medicine, Iksan, Republic of Korea; 3 NADIANBIO Ltd, Wonkwang University School of Medicine, Iksan, Jeonbuk, Republic of Korea; 4 Department of Biochemistry & Molecular Biology, Institute of Medical Science, Jeonbuk National University Medical School, Jeonju, Republic of Korea; 5 Department of Biochemistry, Wonkwang University School of Medicine, Iksan, Republic of Korea; 6 Sarcopenia Total Solution Center, Wonkwang University School of Medicine, Iksan, Republic of Korea; 7 Institute of Wonkwang Medical Science, Wonkwang University, Iksan, Republic of Korea

**Keywords:** dunnione, NAD^+^ metabolism, NETosis, NQO1, psoriasis, redox regulation, SIRT1

## Abstract

**Introduction:**

Neutrophil extracellular traps (NETs) are important effectors of innate immunity but can contribute to inflammatory pathology when dysregulated. Although reactive oxygen species (ROS) are known to promote NET formation, the metabolic mechanisms that regulate NETosis remain incompletely understood. Here, we investigated whether modulation of intracellular NAD⁺/NADH redox balance regulates NET formation and inflammatory responses.

**Methods:**

Using primary neutrophils, differentiated HL-60 cells, and an imiquimod-induced psoriasis-like mouse model, we investigated the effects of dunnione, an NQO1 substrate, on NET formation and inflammatory responses.

**Results:**

Dunnione significantly suppressed NET formation and inflammation. Dunnione increased the intracellular NAD⁺/NADH ratio in an NQO1-dependent manner and was accompanied by increased SIRT1 activity. This effect was associated with suppression of PAD4-mediated histone H3 citrullination and attenuation of NF-κB-dependent pro-inflammatory cytokine expression via deacetylation. In addition, dunnione reduced ROS generation and NOX2 activation, further limiting NET formation. The inhibitory effect of dunnione on NET formation was lost under NQO1-deficient conditions, indicating that NQO1 is required for dunnione responsiveness rather than for NET formation itself. *In vivo*, dunnione significantly attenuated psoriasis-like skin inflammation in association with reduced NET formation and inflammatory responses.

**Discussion:**

Collectively, these findings support a metabolic mechanism in which NQO1-dependent redox modulation, accompanied by NAD^+^SIRT1-associated signaling, contributes to the suppression of NETosis and inflammatory responses.

## 1 Introduction

Neutrophils are essential components of the innate immune system and play a central role in host defense against invading pathogens (1, 2). In addition to phagocytosis and degranulation, activated neutrophils can release neutrophil extracellular traps (NETs), which are composed of decondensed chromatin decorated with histones and antimicrobial proteins (3–6). NET formation is an effective antimicrobial strategy; however, excessive or dysregulated NETosis has been increasingly recognized as a contributor to tissue damage and chronic inflammation (4, 5, 7).

Emerging evidence indicates that NETs act not only as antimicrobial structures but also as potent mediators of inflammatory amplification (4, 7, 8). NET-derived components can function as damage-associated molecular patterns (DAMPs), activating both immune and non-immune cells and promoting the production of pro-inflammatory cytokines (7–9). In chronic inflammatory diseases such as psoriasis, NETs have been implicated in the activation of keratinocytes and the amplification of inflammatory signaling pathways, including NF-κB and IL-17-driven responses (8–13). Despite these advances in inflammatory pathology, the intracellular mechanisms that regulate NET formation and determine its inflammatory potential remain incompletely understood.

Recent studies have highlighted the importance of cellular metabolism in regulating immune cell function, a concept referred to as immunometabolism (14–16). In particular, intracellular redox balance, largely governed by the NAD^+^/NADH ratio, plays a critical role in regulating oxidative stress responses and inflammatory signaling pathways (14, 15, 17). NAD^+^ serves as an essential cofactor for a variety of enzymes involved in cellular homeostasis, including sirtuin family proteins, which link metabolic status to transcriptional and epigenetic regulation (14, 15, 18). Although metabolic regulation has been implicated in immune cell activation, its role in controlling neutrophil activation and NETosis remains to be fully elucidated.

Sirtuin 1 (SIRT1), a NAD^+^-dependent deacetylase, has been shown to regulate inflammatory responses through deacetylation of transcription factors such as NF-κB, thereby suppressing pro-inflammatory gene expression (15, 18–20). In addition to transcriptional regulation, NET formation requires chromatin decondensation, a process mediated by peptidylarginine deiminase 4 (PAD4), which catalyzes histone citrullination (21–24). However, whether metabolic regulation of SIRT1 activity influences PAD4-dependent NET formation and NET-associated inflammatory signaling has not been clearly defined.

Dunnione, a naturally occurring naphthoquinone, is a substrate of NAD(P)H:quinone oxidoreductase 1 (NQO1) and has been reported to modulate intracellular redox balance by increasing the NAD^+^/NADH ratio (25, 26). Activation of the NQO1–NAD^+^–SIRT1 axis has been associated with enhanced SIRT1 activity and suppression of oxidative stress and inflammatory signaling pathways (18, 25, 27). Despite these findings, the potential role of dunnione in regulating neutrophil function and NET formation has not been investigated.

In the present study, we examined whether modulation of NAD^+^-dependent redox metabolism by dunnione regulates NET formation and NET-associated inflammatory responses. Using primary neutrophils, differentiated HL-60 cells, and an imiquimod-induced psoriasis-like mouse model, we show that dunnione suppresses NET formation and inflammatory signaling in an NQO1–dependent manner, with accompanying changes in NAD^+^–SIRT1-associated signaling. Dunnione treatment was also associated with reduced PAD4-mediated histone citrullination and attenuation of NF-κB-dependent cytokine expression together with increased SIRT1 activity; however, the present data do not establish SIRT1 as an indispensable mediator. These findings highlight a metabolic mechanism linking redox regulation to NETosis and suggest a potential therapeutic strategy for inflammatory diseases characterized by excessive NET formation.

## 2 Methods

### 2.1 Ethical approval

All animal experiments were conducted in accordance with institutional guidelines and approved by the Animal Care and Use Committee of Wonkwang University School of Medicine (Approval Nos. WKU22–22 and WKU22-23).

### 2.2 Chemicals

IMQ cream (5%; Aldara^®^; 3M Pharmaceuticals, UK) was used to induce psoriasis-like dermatitis. Hydrogen peroxide (SP-6000) and protein blocking solutions (2.5% horse serum, S-2012-50) were obtained from Vector Laboratories (USA).

Dunnione was provided by NADIANBIO Ltd. (Iksan, Republic of Korea) and dissolved in corn oil for *in vivo* administration or in RPMI-1640 medium for *in vitro* experiments.

The primary antibodies used included anti-CD4 (1:300, ab183685), anti-H3cit (1:300, ab219407), anti-PAD4 (1:300, ab96758) (all from Abcam, UK), anti-MPO (1:300, AF3667; R&D Systems, USA), anti-phosphorylated p40-phox (1:300, sc-33403; Santa Cruz Biotechnology, USA), anti-acetyl-NF-κB (1:500, PA5-17264; Invitrogen, USA), anti-NQO1 (1:1000, sc-32793; Santa Cruz Biotechnology), anti-NF-κB (PA1-186; Invitrogen), and anti-β-actin (1:1000, sc-4778; Santa Cruz Biotechnology). Alexa Fluor 488- and 568-conjugated secondary antibodies (Invitrogen) were used at a 1:1000 dilution. HRP-conjugated secondary antibodies were purchased from Bethyl Laboratories (A120-101P; USA). NET formation was quantified using the Quant-iT PicoGreen dsDNA assay kit (P11496; Invitrogen). Cytokine profiling was performed using a Mouse XL Cytokine Array Kit (ARY028; R&D Systems). RPMI-1640 medium and HEPES buffer (25 mM; Gibco, USA) were used for cell culture. Percoll (P1644; Sigma-Aldrich, USA) and ACK lysis buffer (Gibco) were used for neutrophil isolation. SIRT1 activity was measured using a commercial assay kit (ab156065; Abcam). ROS levels were detected using CM-H_2_DCFDA (C6827; Invitrogen), and the NAD^+^/NADH ratio was measured using a fluorometric assay kit (E2ND-100; BioAssay Systems, USA).

### 2.3 Cell culture and differentiation

HL-60 cells obtained from the Korean Cell Line Bank were cultured in RPMI-1640 medium supplemented with 10% fetal bovine serum, antibiotics, and 25 mM HEPES at 37 °C in a humidified atmosphere containing 5% CO_2_.

Differentiation into neutrophil-like cells was induced by treatment with 1.25% DMSO for 5 days. Differentiation efficiency was assessed using CD11b expression analyzed on a FACSymphony A3 flow cytometer (BD Biosciences) and Wright–Giemsa staining cells were observed under a ZEISS microscope.

### 2.4 Animal model

Female C57BL/6 mice (7–8 weeks old) were obtained from Nara Biotech (Seoul, Korea). C57BL/6 NQO1^-^/^-^ mice were provided by KRIBB (Daejeon, Korea). Animals were housed under specific pathogen-free conditions.

Mice were randomly assigned to the control, dunnione alone, IMQ alone, IMQ plus dunnione (20 mg/kg), and IMQ plus dunnione (40 mg/kg) groups (n = 6 per group). Psoriasis-like dermatitis was induced by daily topical application of IMQ cream (62.5 mg, 5%) to shaved dorsal skin for five consecutive days. Dunnione was orally administered once daily, starting 2 days before IMQ treatment, and continued for 7 days. Disease severity was evaluated daily using the modified Psoriasis Area and Severity Index (PASI) scoring system (28).

### 2.5 Neutrophil isolation

Bone marrow neutrophils were isolated using a discontinuous Percoll gradient (65%/78%). Cell purity was determined by flow cytometric analysis of CD11b^+^Ly6G^+^ populations using a FACSymphony A3 flow cytometer (BD Biosciences) and viability was assessed by fluorescence-based cell counting.

### 2.6 *In vitro* NET induction

The differentiated HL-60 (dHL-60) cells and primary neutrophils were stimulated with phorbol 12-myristate 13-acetate (PMA; 100 nM) in the presence or absence of dunnione (0.5 μM). ROS levels and NAD^+^/NADH were measured at 30 min, and NET formation was assessed after 4 h.

### 2.7 NET immunostaining

Cells were fixed in 4% paraformaldehyde, permeabilized, blocked, and incubated with antibodies against MPO, H3cit, PAD4, phosphorylated p40-phox, and acetylated NF-κB. After secondary antibody incubation and DAPI staining, images were obtained using ZEISS confocal microscopy and analyzed with Zen software.

### 2.8 Histological analysis

Skin tissues were fixed in 10% neutral buffered formalin, paraffin-embedded, sectioned at 4 µm, and stained with hematoxylin and eosin (H&E). The epidermal thickness was quantified in five randomly selected fields per section using a ZEISS Axio Scan Z1 microscope.

### 2.9 Immunofluorescence staining

Paraffin-embedded skin sections (4 µm) were deparaffinized, rehydrated, and subjected to antigen retrieval (110 °C, 18 min). Endogenous peroxidase activity was blocked, followed by blocking of nonspecific binding using 2.5% horse serum. Sections were incubated overnight at 4 °C with primary antibodies against CD4, H3cit, MPO, and PAD4 (all 1:300). After washing, Alexa Fluor 488- or 568-conjugated secondary antibodies (1:1000) were applied, and the nuclei were counterstained with DAPI. Images were acquired using the ZEISS Axio Scan system.

### 2.10 Hematological analysis

Whole blood samples were collected into EDTA-coated tubes and analyzed using an automated hematology analyzer (XN-1000; Sysmex, Kobe, Japan) to determine leukocyte and neutrophil counts.

### 2.11 MPO–DNA complex ELISA

Plasma NET levels were quantified using a capture ELISA. Plates were coated overnight with anti-MPO antibody (5 μg/mL). After blocking, plasma samples diluted in Tris-EDTA buffer were incubated for 2 h at 37 °C. DNA content was detected using PicoGreen reagent and fluorescence was measured at 485/535 nm using a microplate reader (Synergy H1, BioTek, VT, USA).

### 2.12 Cytokine array analysis

Plasma cytokine profiles were assessed using a Mouse XL Cytokine Array Kit (R&D Systems). Membranes were incubated with plasma samples overnight, followed by detection with the antibody cocktail and streptavidin-HRP. Signal intensities were quantified using ImageJ software. The cytokine-array analysis was performed using two biological replicates per group (n=2) and was therefore interpreted as an exploratory, hypothesis-generating analysis.

### 2.13 Western blotting

Skin tissues and dHL-60 cells were lysed in RIPA buffer. Proteins were separated using SDS-PAGE, transferred to nitrocellulose membranes, and probed with antibodies against NQO1, acetyl-NF-κB, total NF-κB, and β-actin. Signals were visualized using chemiluminescence and quantified using densitometry.

### 2.14 SIRT1 activity assay

SIRT1 enzymatic activity was measured using a commercial fluorescence-based assay kit according to the manufacturer’s instructions. Fluorescence was detected at excitation and emission wavelengths of 350/450 nm using a microplate reader (Synergy H1, BioTek).

### 2.15 ROS measurement

Intracellular ROS levels were assessed using CM-H_2_DCFDA (5 μM). After staining for 30 min at 37 °C, cells were washed and analyzed using flow cytometry with a FACSymphony A3 flow cytometer (BD Biosciences).

### 2.16 NAD^+^/NADH ratio measurement

The NAD^+^/NADH ratio was determined using a fluorometric cycling assay kit. The cell lysates were processed according to the manufacturer’s instructions and analyzed using a microplate reader (Synergy H1, BioTek).

### 2.17 Quantitative real-time PCR

Total RNA was extracted using TRIzol reagent and reverse-transcribed into cDNA. Quantitative real-time PCR (qRT-PCR) was performed using SYBR Green on a CFX Connect Real-Time PCR System (Bio-Rad). Relative gene expression levels were calculated using the 2^-ΔΔCt^ method with β-actin as the internal reference gene. The primer sequences are listed in **Supplementary Table S1**.

### 2.18 Statistical analysis

Data are presented as mean ± SD. Statistical analyses were performed using GraphPad Prism 10. One-way ANOVA was used for multiple comparisons, with p-values < 0.05 considered statistically significant.

## 3 Results

### 3.1 Dunnione alleviates IMQ-induced psoriasis-like inflammation in C57BL/6 mice

Topical application of IMQ is widely recognized as a robust murine model that recapitulates the clinical and histopathological features of human psoriasis (29). To evaluate the therapeutic potential of dunnione, an IMQ-induced psoriasis-like mouse model was established using C57BL/6 wild-type mice. IMQ was topically applied for five consecutive days, whereas dunnione was orally administered 2 days prior to IMQ treatment and continued for 7 days (**Figure 1A**). IMQ application resulted in a significant reduction in body weight compared to that in both the normal control and dunnione-only groups. Notably, co-administration of dunnione dose-dependently mitigated IMQ-induced body weight loss, suggesting a protective effect of dunnione on systemic health (**Figure 1B**).

**Figure 1:**
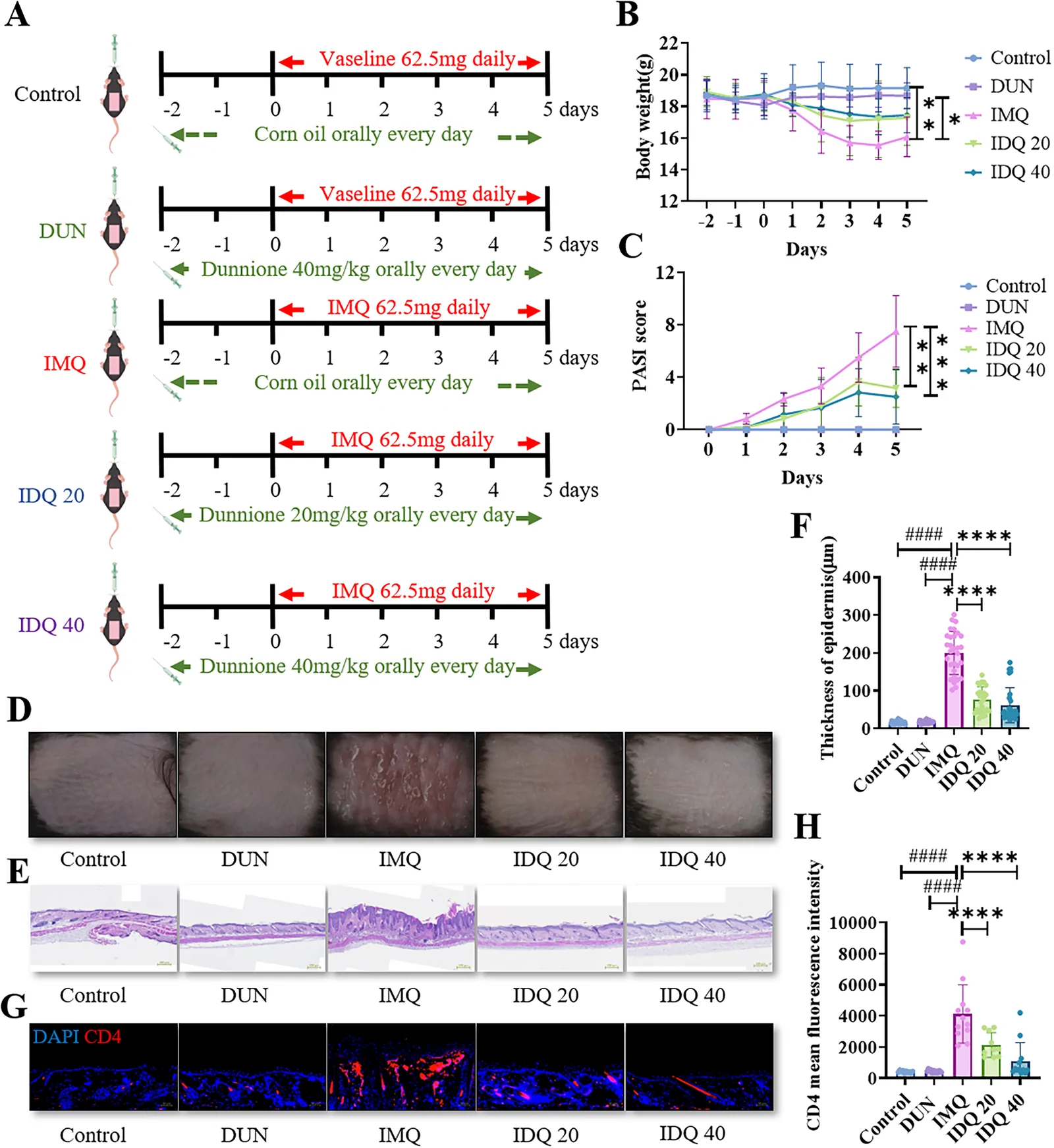
Dunnione attenuates IMQ-induced psoriasis-like inflammation in C57BL/6 mouse model. **(A)** Experimental schedule of the animal study. **(B)** Changes in body weight during the experimental period. **(C)** Daily assessment of Psoriasis Area and Severity Index (PASI) scores. **(D)** Representative macroscopic images of dorsal skin lesions on Day 5. **(E)** Representative H&E-stained sections of dorsal skin. **(F)** Quantification of epidermal thickness from H&E-stained sections. **(G)** Immunofluorescence **(IF)** staining of CD4 (red) and DAPI (blue) in skin sections. **(H)** Representative images and quantitative analysis of CD4^+^ T-cell infiltration in skin. Data are presented as mean ± SD (n = 6 per group). Statistical analysis was performed using one-way ANOVA. *P < 0.05, **P < 0.01, ***P < 0.001, ****P < 0.0001, ^####^P < 0.0001. H&E, hematoxylin and eosin; IMQ, imiquimod.

The severity of psoriatic inflammation was evaluated daily using the PASI, which includes erythema, scaling, and thickening scores (28). PASI scores remained consistently low in the control and dunnione-only groups, whereas IMQ treatment induced a progressive increase that reached a significant peak on day 5. Dunnione co-treatment significantly reduced the PASI scores compared with those in the IMQ only group, with a more pronounced improvement observed at higher dunnione doses (**Figure 1C**). Consistently, the IMQ-treated mice developed visible psoriasis-like lesions characterized by erythema, scaling, and epidermal thickening. Dunnione administration markedly alleviated these symptoms, particularly at 40 mg/kg (**Figure 1D**).

Histopathological analysis confirmed that IMQ treatment induced marked epidermal hyperplasia, hyperkeratosis, and dense inflammatory cell infiltration into the dermis. Dunnione treatment substantially ameliorated these pathological alterations by restoring epidermal integrity, reducing keratosis (**Figure 1D, E**), and decreasing epidermal thickness in a dose-dependent manner (**Figure 1F**). Moreover, IF staining for CD4^+^ T cells revealed pronounced infiltration in IMQ-treated lesions, which was significantly reduced by both dunnione treatments, with more robust suppression observed at 40 mg/kg (**Figure 1G, H**).

Collectively, these results demonstrate that dunnione exerts potent, dose-dependent therapeutic effects on IMQ-induced psoriasis-like inflammation by preserving systemic health, alleviating clinical and histopathological severity, and reducing immune cell infiltration.

### 3.2 Dunnione attenuates NET formation and NF-κB-mediated inflammation in an IMQ-induced psoriasis-like mouse model

Excessive NET formation contributes to the exacerbation of psoriasis, whereas suppression of NET formation alleviates psoriatic inflammation (8). To investigate the relationship between psoriasis and NETs, peripheral blood neutrophil count and plasma NET levels were measured. Both parameters were significantly elevated in IMQ-treated mice compared with those in controls, whereas dunnione administration dose-dependently reduced these increases (**Figure 2A, B**). Total leukocyte and monocyte count exhibited a similar trend, whereas lymphocytes showed the opposite pattern compared with neutrophils (**Supplementary Figure S1**). Consistently, the expression of E-selectin, an adhesion molecule critical for neutrophil recruitment, was upregulated in IMQ-treated mice, but significantly downregulated by dunnione treatment in a dose-dependent manner (**Supplementary Figure S2**). To assess inflammatory activation in skin lesions, both acetylated NF-κB (acetyl-NF-κB) and total NF-κB expression were analyzed. IMQ treatment markedly increased acetylated-NF-κB, the transcriptionally active form, whereas dunnione treatment dose-dependently reduced its acetylation, indicating suppression of NF-κB-mediated inflammatory signaling. Total NF-κB expression remained elevated in both the IMQ- and dunnione-treated groups, with no significant differences between them (**Figures 2C–E**). To examine changes in the distribution of NETs in inflamed skin, we performed IF co-staining using H3cit and MPO as NET markers. Marked NET formation was observed at the dermal-epidermal junctions in the IMQ-treated group. In contrast, co-treatment with dunnione markedly reduced NET accumulation, which was more pronounced in the 40 mg/kg dunnione-treated group (**Figures 2F–H**). Similarly, the expression of PAD4, a key enzyme responsible for histone H3 citrullination and NET formation, was significantly elevated in IMQ-treated mice but dose-dependently suppressed by dunnione (**Figure 2I, J**).

**Figure 2:**
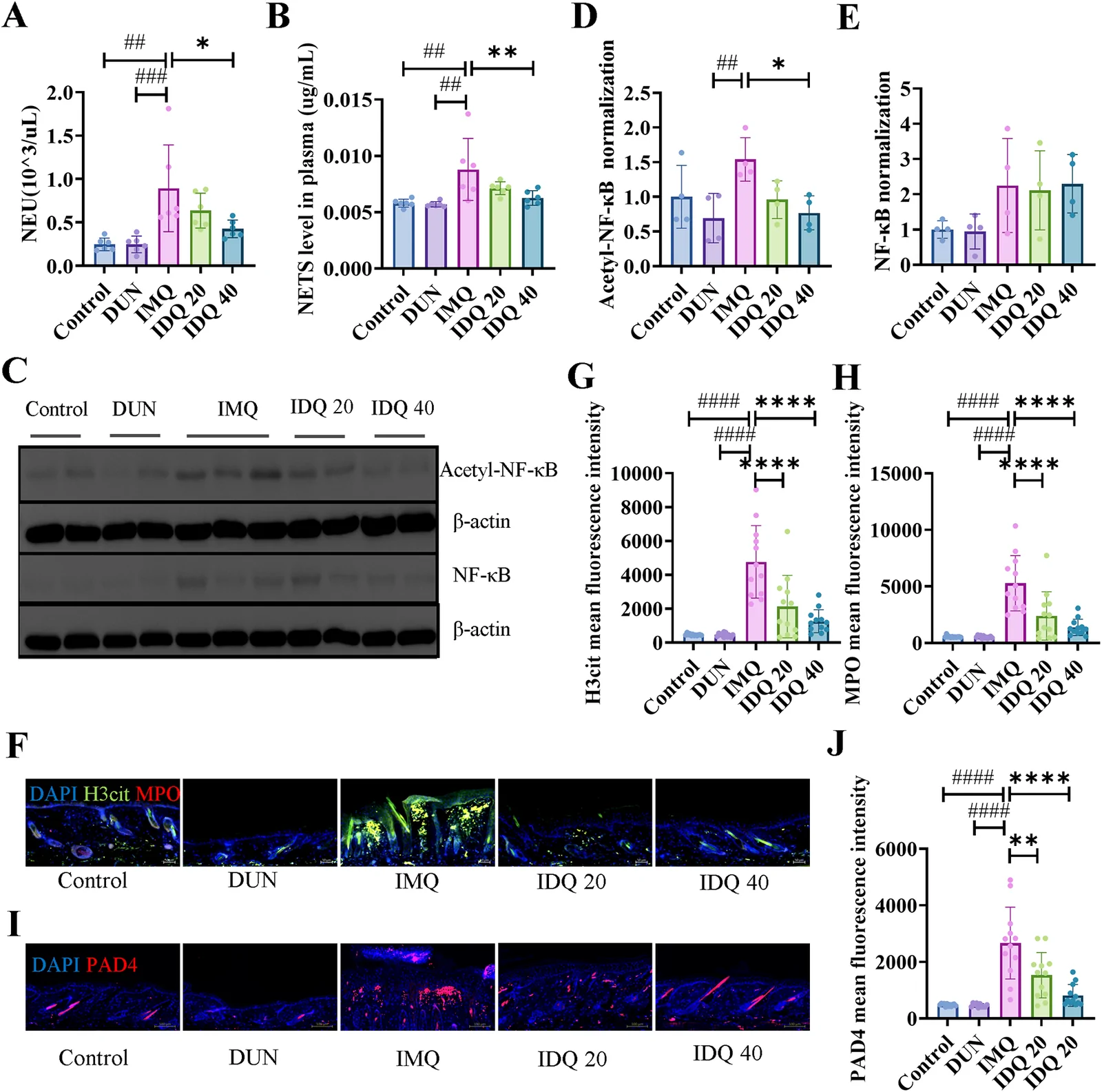
Dunnione attenuates NET-associated inflammatory responses in IMQ-induced psoriasis-like mouse model. **(A)** Peripheral blood neutrophil counts. **(B)** Plasma NET levels measured using MPO–DNA enzyme-linked immunosorbent assay. **(C)** Western blot analysis of acetylated NF-κB (acetyl-NF-κB) and total NF-κB expression. **(D, E)** Quantification of acetyl-NF-κB and total NF-κB protein levels. **(F)** IF co-staining of MPO (red), H3cit (green), and DAPI (blue) to assess NET formation in mouse skin sections. **(G, H)** Representative images and quantitative analysis of H3cit and MPO expression in skin. **(I)** IF staining of PAD4 (red) and DAPI (blue) from mouse skin sections. **(J)** Representative images and quantitative analysis of PAD4 expression in skin. Data are presented as mean ± SD (n = 6 per group). Statistical analysis was performed using one-way ANOVA. *P < 0.05, **P < 0.01, ****P < 0.0001, ^##^P < 0.01, ^###^P < 0.001, ^####^P < 0.0001. IF, immunofluorescence; IMQ, imiquimod; MPO, myeloperoxidase; NET, neutrophil extracellular trap.

These findings indicate that dunnione alleviates psoriatic inflammation by reducing PAD4 expression, inhibiting neutrophil infiltration, suppressing NET formation, and attenuating NF-κB-dependent inflammatory signaling.

### 3.3 Dunnione attenuates PMA-induced NET-associated responses through NQO1-associated redox modulation

To investigate the direct effects of dunnione on NET formation *in vitro*, primary mouse bone marrow neutrophils were isolated using 65% and 78% Percoll gradients to achieve 96% purity, as confirmed using flow cytometry (**Figure 3A**). NET formation was induced by PMA. IF co-staining of H3cit, MPO, and DAPI revealed abundant NET structures in PMA-stimulated neutrophils, whereas co-treatment with dunnione significantly reduced NET formation (**Figures 3B–E**).

**Figure 3:**
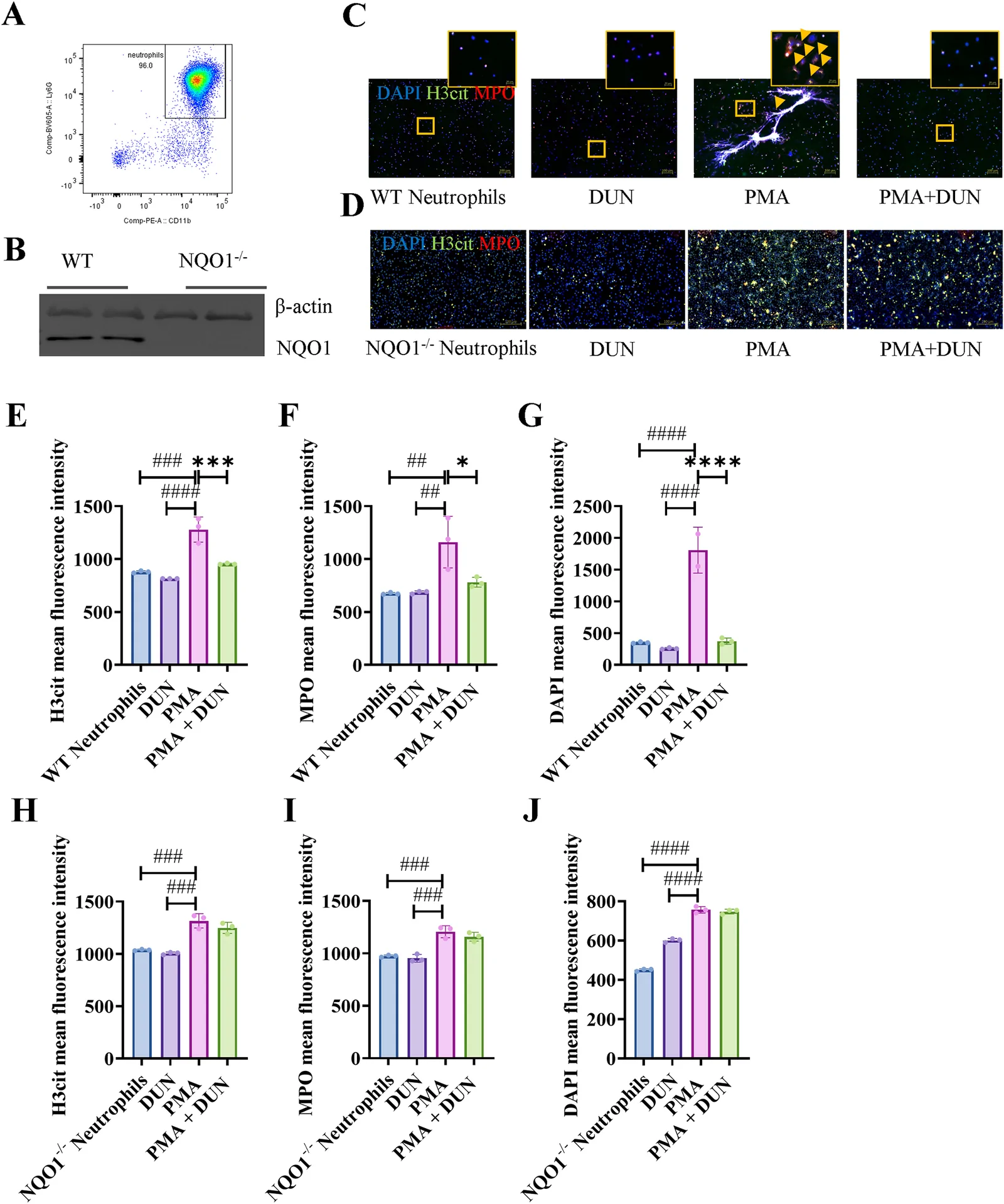
Dunnione suppresses PMA-induced NET formation through NQO1-associated redox modulation. **(A)** Flow cytometric analysis of CD11b and Ly6G expression to assess the purity of Percoll-isolated bone marrow neutrophils from wild-type mice. **(B)** Western blotting analysis of NQO1 expression in mouse skin tissues. **(C)** IF co-staining of MPO (red), H3cit (green), and DAPI (blue) to visualize NET-associated structures in PMA-stimulated wild-type neutrophils. **(D)** IF co-staining of MPO (red), H3cit (green), and DAPI (blue) to assess NET-associated structures in PMA-stimulated neutrophils from NQO1⁻/⁻ mice. **(E–G)** Representative images and quantitative analysis of H3cit, MPO, and DAPI fluorescence intensity in wild-type neutrophils. **(H–J)** Representative images and quantitative analysis of H3cit, MPO, and DAPI fluorescence intensity in NQO1⁻/⁻ neutrophils. Data are presented as mean ± SD (n = 3 per group). Statistical analysis was performed using one-way ANOVA. *P < 0.05, ***P < 0.001, ****P < 0.0001, ^##^P < 0.01, ^###^P < 0.001, ^####^P < 0.0001. IF, immunofluorescence; MPO, myeloperoxidase; NET, neutrophil extracellular trap; PMA, phorbol 12-myristate 13-acetate.

Dunnione acts as a substrate for NQO1, therefore, we evaluated whether this effect occurred with NQO1 activation. In neutrophils derived from NQO1-knockout mice (**Figure 3F**), PMA stimulation substantially induced NET formation, whereas dunnione co-treatment failed to suppress it (**Figures 3G–J**). These findings demonstrated that NQO1 is required for the inhibitory response to dunnione. Importantly, NQO1 deficiency did not increase PMA-induced NET formation under the conditions tested, suggesting that PMA-induced NETosis can proceed through NQO1-independent mechanisms.

### 3.4 Dunnione modulates NOX2-associated ROS production and cellular redox balance in dHL-60 cells

Owing to the limited number of primary neutrophils obtainable from the bone marrow (30), we used the HL-60 cell line as a model to study NET formation (31). HL-60 cells were differentiated into neutrophil-like cells (dHL-60) using 1.25% DMSO treatment for 5 days. Flow cytometry of CD11b expression confirmed a differentiation efficiency of 94.6% (**Figure 4A**) and morphological analysis revealed indented or horseshoe-shaped nuclei, which are characteristic of mature neutrophils (**Figure 4B**).

**Figure 4:**
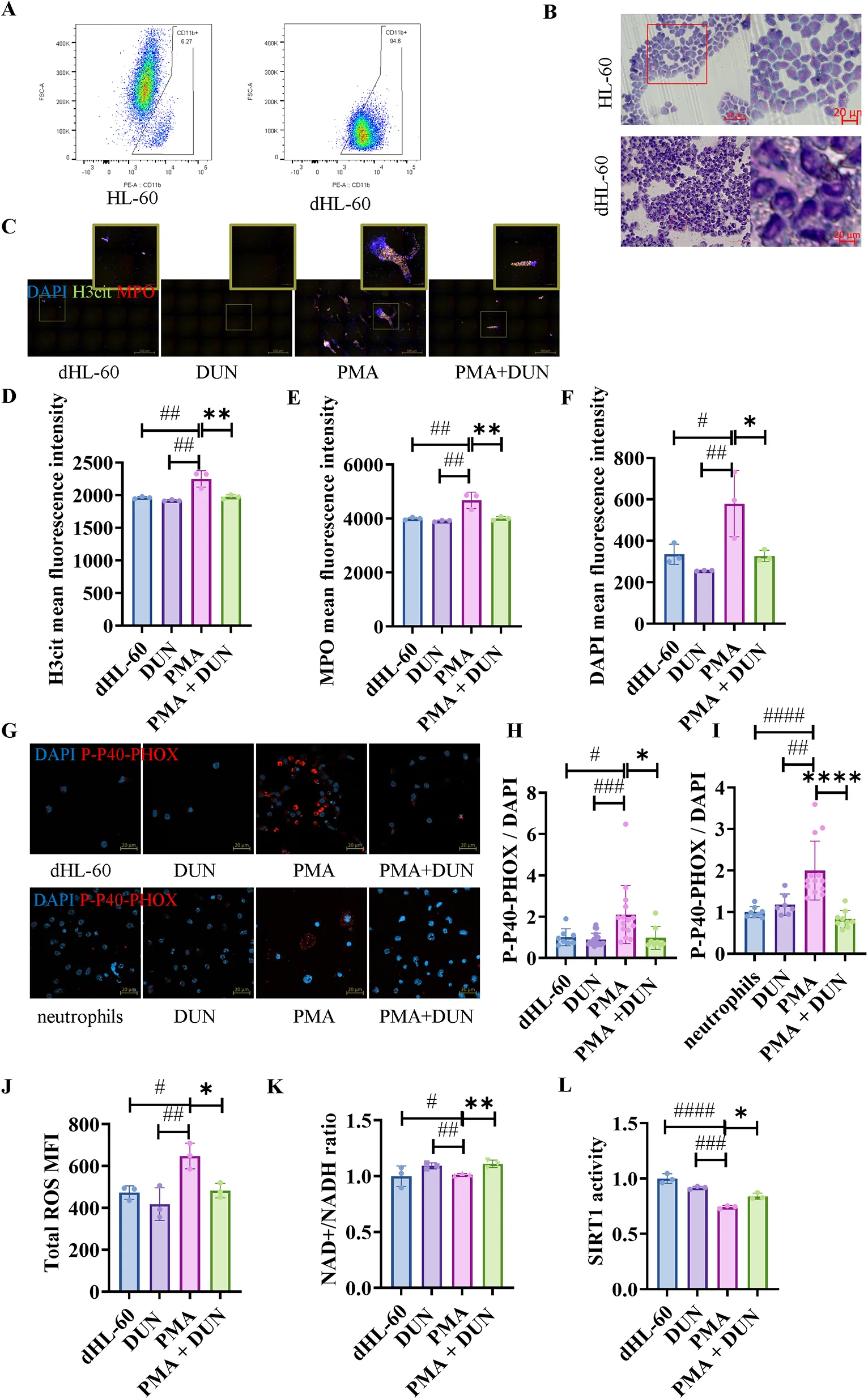
Dunnione limits NOX2-associated ROS production and modulates redox balance in dHL-60 cells. **(A)** Flow cytometric analysis of CD11b expression to assess the differentiation of HL-60 cells into neutrophil-like dHL-60 cells. **(B)** Wright–Giemsa staining showing the morphological features of HL-60 and dHL-60 cells. **(C)** IF co-staining of MPO (red), H3cit (green), and DAPI (blue) to visualize NET-associated structures in PMA-stimulated dHL-60 cells. **(D–F)** Representative images and quantitative analysis of H3cit, MPO, and DAPI fluorescence intensity in dHL-60 cells. **(G)** IF staining of phosphorylated p40-phox (p-p40-phox, red) and DAPI **(blue)** in dHL-60 cells and primary bone marrow neutrophils. **(H, I)** Representative images and quantitative analysis of p-p40-phox expression in dHL-60 cells and bone marrow neutrophils. **(J)** Flow cytometric analysis of intracellular ROS levels using CM-H_2_DCFDA staining. **(K)** Measurement of the NAD^+^/NADH ratio in dHL-60 cells. **(L)** SIRT1 activity in dHL-60 cells following PMA and dunnione treatment. Data are presented as mean ± SD (n ≥ 3 per group). Statistical analysis was performed using one-way ANOVA. *P < 0.05, **P < 0.01, ****P < 0.0001, ^#^P < 0.05, ^##^P < 0.01, ^###^P < 0.001, ^####^P < 0.0001. IF, immunofluorescence; MPO, myeloperoxidase; NET, neutrophil extracellular trap; PMA, phorbol 12-myristate 13-acetate; ROS, reactive oxygen species.

Following PMA stimulation, IF co-staining with H3cit, MPO, and DAPI confirmed robust NET formation in dHL-60 cells, which was markedly suppressed by dunnione co-treatment (**Figures 4C–F**). PMA-induced NET formation occurs through the activation of the NOX2-dependent ROS pathway, in which phosphorylation of the NOX2 subunit p40-phox plays a critical regulatory role (32, 33). IF staining showed that PMA significantly increased phosphorylated p40-phox (p-p40-phox) expression, whereas dunnione co-treatment notably reduced its phosphorylation in both dHL-60 cells and primary neutrophils (**Figures 4G–I**). Consistently, flow cytometry using CM-H_2_DCFDA revealed that PMA markedly increased intracellular ROS levels, which were effectively reduced by dunnione treatment (**Figure 4J**).

NOX2-derived ROS generation affects the cellular redox state, therefore, the NAD^+^/NADH ratio was measured. PMA treatment significantly decreased the NAD^+^/NADH ratio, indicating redox imbalance, whereas dunnione restored this ratio toward control levels (**Figure 4K**). These findings indicated that dunnione suppressed NET formation by inhibiting NOX2 activation and maintaining NQO1-mediated redox homeostasis.

### 3.5 Dunnione enhances SIRT1 activity to attenuate PAD4-induced H3cit formation and NF-κB-driven cytokine expression

NAD^+^ is an essential co-substrate for the deacetylase SIRT1. PMA stimulation markedly reduced SIRT1 activity, whereas co-treatment with dunnione significantly restored SIRT1 levels (**Figure 4L**). SIRT1 is an endogenous suppressor of PAD4, a critical enzyme for histone H3 citrullination, therefore, we investigated PAD4 expression. IF staining demonstrated that PMA markedly increased PAD4 expression in both dHL-60 cells and primary neutrophils, whereas dunnione co-treatment significantly suppressed PAD4 levels (**Figures 5A–C**). This trend was consistent with the previously observed reduction in H3cit levels, supports an association between increased SIRT1 activity and reduced PAD4/H3cit responses during dunnione-mediated NET inhibition.

**Figure 5:**
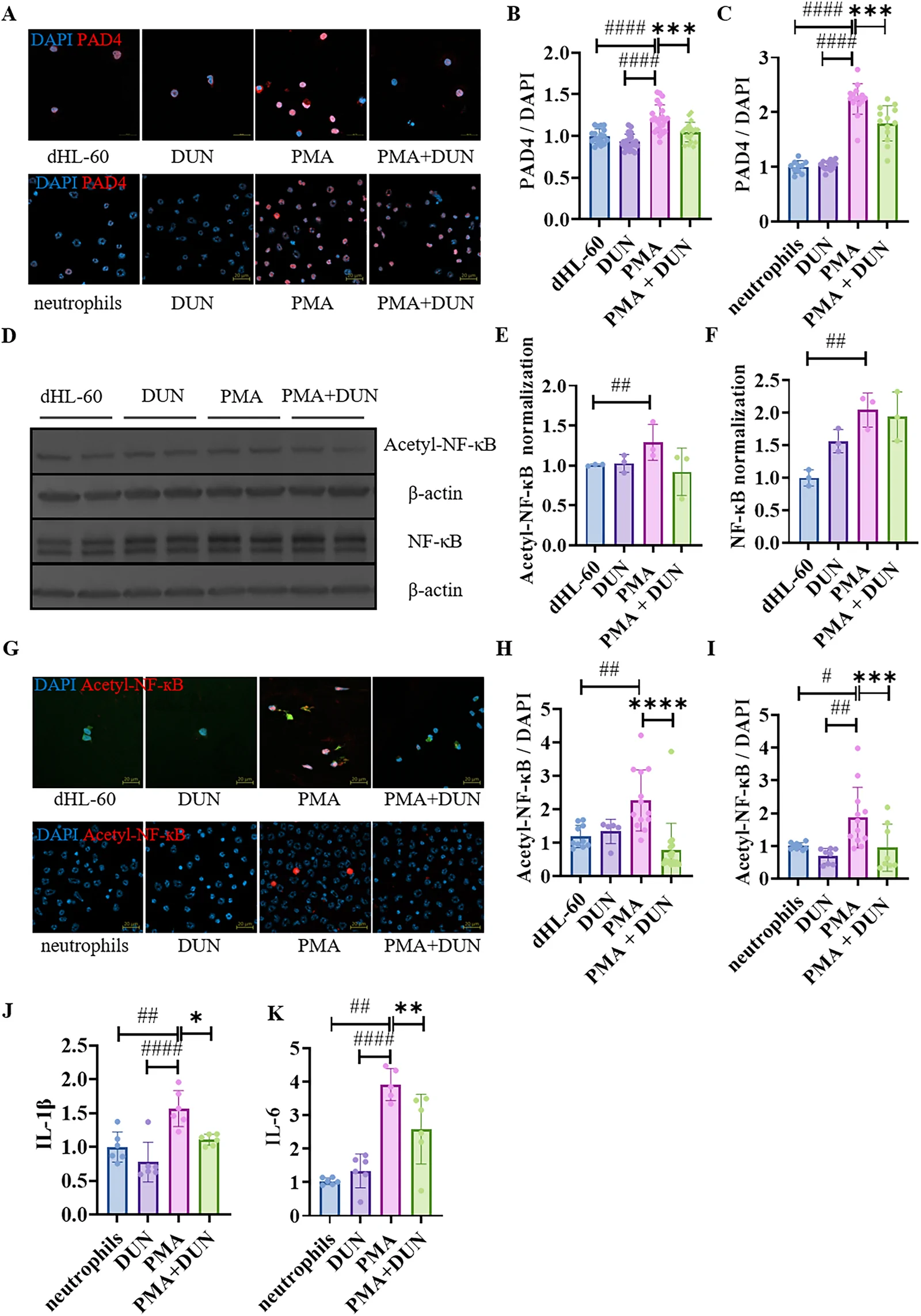
Dunnione modulates SIRT1-associated signaling linked to PAD4-dependent histone citrullination and inflammatory gene expression. **(A)** IF staining of PAD4 (red) and DAPI (blue) in neutrophil-like dHL-60 cells and primary bone marrow neutrophils. **(B, C)** Representative images and quantitative analysis of the PAD4 signal intensity in neutrophil-like dHL-60 cells and bone marrow neutrophils. **(D)** Western blot analysis of acetyl-NF-κB and total NF-κB expression. **(E, F)** Quantification of acetyl-NF-κB and total NF-κB protein levels. **(G)** IF staining of acetyl-NF-κB (red) and DAPI (blue) in neutrophil-like dHL-60 cells and bone marrow neutrophils. **(H, I)** Representative images and quantitative analysis of normalized acetyl-NF-κB signal intensity in neutrophil-like dHL-60 cells and bone marrow neutrophils. **(J, K)** Relative mRNA expression levels of IL-1β and IL-6 in PMA-activated wild-type bone marrow neutrophils. Data are presented as mean ± SD (n ≥ 5 per group; for western blotting, n = 3; for qPCR, n = 6 per group). Statistical analysis was performed using one-way ANOVA. *P < 0.05, **P < 0.01, ***P < 0.001, ****P < 0.0001, ^#^P < 0.05, ^##^P < 0.01, ^####^P < 0.0001. IF, immunofluorescence; PMA, phorbol 12-myristate 13-acetate.

SIRT1 also acts as an NAD-dependent deacetylase that suppresses inflammatory signaling by deacetylating transcription factors, such as NF-κB. PMA stimulation decreased SIRT1 activity while increasing acetyl-NF-κB. Co-treatment with dunnione effectively reversed this trend, reducing NF-κB acetylation without affecting total NF-κB protein levels (**Figures 5D–F**). IF staining further confirmed that PMA significantly enhanced acetyl-NF-κB expression, which was attenuated by dunnione in both dHL-60 cells and primary neutrophils (**Figures 5G–I**).

Acetyl-NF-κB functions as a transcriptionally active proinflammatory factor that upregulates cytokines, such as IL-1β and IL-6, therefore, we evaluated these cytokines in neutrophils. PMA stimulation markedly increased *IL-1β* and *IL-6* mRNA levels, whereas co-treatment with dunnione significantly reduced their levels compared with PMA alone (**Figures 5J, K**).

Collectively, these findings show that dunnione enhances SIRT1 activity in parallel with reduced NF-κB acetylation, PAD4 expression, and histone H3 citrullination. These coordinated changes are consistent with a contribution of NAD^+^-SIRT1-associated signaling to NET suppression and inflammatory regulation, although SIRT1 dependency was not directly tested.

## 4 Discussion

In the present study, we demonstrate that modulation of intracellular redox metabolism by dunnione suppresses neutrophil extracellular trap (NET) formation and attenuates inflammatory responses. Mechanistically, our findings support a model in which NQO1–dependent redox modulation is accompanied by restoration of NAD^+^–SIRT1-associated signaling, reduced PAD4-mediated histone citrullination and suppression of NF-κB–dependent cytokine expression. The current data support pathway association but do not establish an obligatory linear NQO1-NAD^+^-SIRT1 signaling axis.

Neutrophils are key effectors of innate immunity, and their activation is tightly associated with the generation of reactive oxygen species (ROS) and inflammatory signaling pathways (17, 34, 35). NF-κB is a central regulator of inflammatory responses, and its activation is closely linked to oxidative stress and cytokine production (17, 19). In addition, SIRT1 has been shown to negatively regulate NF-κB–dependent transcription through deacetylation, thereby modulating inflammatory responses (18, 19). Consistent with these established mechanisms, our data suggest that restoration of NAD^+^–SIRT1-associated signaling may contribute to suppression of NF-κB–driven cytokine expression.

NET formation represents a specialized antimicrobial mechanism in which neutrophils release chromatin structures decorated with antimicrobial proteins (3–5). While NETs are essential for host defense, excessive NETosis contributes to tissue damage and chronic inflammation (4, 5, 7). The formation of NETs is highly dependent on ROS generation through NADPH oxidase activity (34, 36), as well as chromatin decondensation processes involving histone modification (21, 22). In particular, PAD4-mediated histone citrullination is a critical step in NET formation (21, 22). In agreement with these findings, our results show that dunnione treatment suppresses histone citrullination, suggesting that metabolic regulation can influence key steps of NETosis (21, 27, 37, 38).

Psoriasis is a chronic inflammatory disease characterized by dysregulated immune responses and keratinocyte hyperproliferation (39–41). The imiquimod-induced mouse model has been widely used to recapitulate IL-23/IL-17–mediated inflammatory pathways observed in human psoriasis (29, 42). In this context, neutrophils and NETs have emerged as important contributors to disease pathogenesis (8, 10, 43). NET formation is increased in psoriatic lesions and can induce inflammatory responses in keratinocytes, including the production of antimicrobial peptides and cytokines (8, 9, 44). Furthermore, NET-associated cytokines such as IL-17 and IL-22 can amplify local immune responses and sustain chronic inflammation (11, 12, 35). Our findings are consistent with these observations, as dunnione-mediated suppression of NET formation was associated with reduced inflammatory signaling and decreased CD4^+^ T cell infiltration in a psoriasis-like model (8, 25, 37). However, because Th17 differentiation, IL-17A production, and T-cell-intrinsic responses were not directly examined, these data do not establish a direct effect of dunnione on Th17 cells. Emerging evidence highlights the importance of immunometabolism in regulating immune cell function. NAD^+^ metabolism plays a central role in maintaining redox homeostasis and regulating signaling pathways involved in inflammation and aging (14, 15). NAD^+^ also serves as a cofactor for enzymes involved in epigenetic regulation, including SIRT1, which controls histone acetylation and transcriptional activity (18, 20). Recent studies have further demonstrated that SIRT1 functions as an endogenous inhibitor of NETosis, although its activity may be impaired under pathological conditions such as diabetes (38). In line with these reports, dunnione treatment restored the NAD^+^/NADH ratio and SIRT1 activity in parallel with reduced NET formation and inflammatory signaling, supporting a potential contribution of SIRT1-associated signaling without establishing pathway necessity.

Dunnione is a substrate of NQO1 and has been shown to modulate intracellular NAD^+^ levels and redox balance (25, 26). The interaction between NQO1 and SIRT1 has been reported to support SIRT1 function, providing a mechanistic link between redox metabolism and inflammatory regulation (27). Importantly, our previous study demonstrated that the protective effect of dunnione on imiquimod-induced psoriasis-like dermatitis was abolished in NQO1^-/-^ mice, in which dunnione failed to reduce PASI scores, ear thickness, or the expression of psoriatic cytokines (IL-17, IL-22, and IL-23) (25). Likewise, dunnione failed to attenuate imiquimod-induced psoriasis-like phenotypes in skin-specific SIRT1^-/-^ mice (25). These genetic loss-of-function data support the *in vivo* requirement of both NQO1 and SIRT1 for the anti-inflammatory effect of dunnione. Our study extends these findings by demonstrating that dunnione directly regulates neutrophil function and NET formation and that this inhibitory response is lost in NQO1-deficient neutrophils. Notably, NQO1 is required for dunnione responsiveness rather than being universally required for NETosis.

In addition to ROS-dependent mechanisms, NET formation has also been linked to autophagy and other intracellular pathways (36, 45). Moreover, cytokine networks involving IL-6 and other inflammatory mediators play important roles in the transition and amplification of immune responses during inflammation (46). These findings suggest that multiple regulatory layers contribute to NETosis and inflammatory signaling. Although our study focuses on the NAD^+^–SIRT1-associated signaling, additional pathways, including ROS-dependent mechanisms, and cytokine-mediated feedback loops, may also contribute to the effects of dunnione.

Despite these findings, several limitations should be considered. First, although SIRT1 activity was restored by dunnione and was accompanied by reduced PAD4/H3cit responses and NF-κB acetylation, SIRT1 was not directly manipulated in neutrophils in the present study; therefore, SIRT1 cannot be considered an indispensable mediator on the basis of the present data. Direct molecular interactions between SIRT1 and PAD4 also remain to be fully elucidated. Second, although the loss of dunnione-mediated NET suppression in NQO1-deficient neutrophils supports an NQO1-dependent cellular response, NET-specific readouts were not directly measured in NQO1-deficient mice *in vivo*. Third, the present study did not directly examine Th17 differentiation, IL-17A production, RORγt expression, or T-cell-intrinsic responses; therefore, the reduced CD4+ T-cell infiltration should not be interpreted as evidence of a direct anti-Th17 effect. Fourth, the relative contribution of ROS modulation versus NAD^+^-dependent signaling requires further investigation. Fifth, the cytokine-array/pathway-enrichment analysis in **Supplementary Figure S2** was exploratory because it was based on a limited number of biological samples (n = 2 per group). Finally, while the imiquimod-induced model captures key features of psoriasis and HL-60-derived neutrophil-like cells are widely used experimentally, neither model fully recapitulates the complexity of human disease or primary neutrophil biology (30, 31).

In conclusion, our study identifies NQO1-dependent redox modulation as an important component of dunnione-mediated suppression of NET formation and inflammatory signaling. The accompanying restoration of the NAD^+^/NADH ratio and SIRT1 activity, together with reduced PAD4/H3cit responses and NF-κB acetylation, supports a contributory role for NAD^+^-SIRT1–associated signaling, although its causal necessity remains to be established. These findings provide new insights into metabolic regulation of NETosis and support further evaluation of redox-targeted strategies for inflammatory diseases characterized by excessive NET formation. These findings provide a rationale for the further exploration of NQO1-dependent redox modulation as a therapeutic target (**Figure 6**).

**Figure 6:**
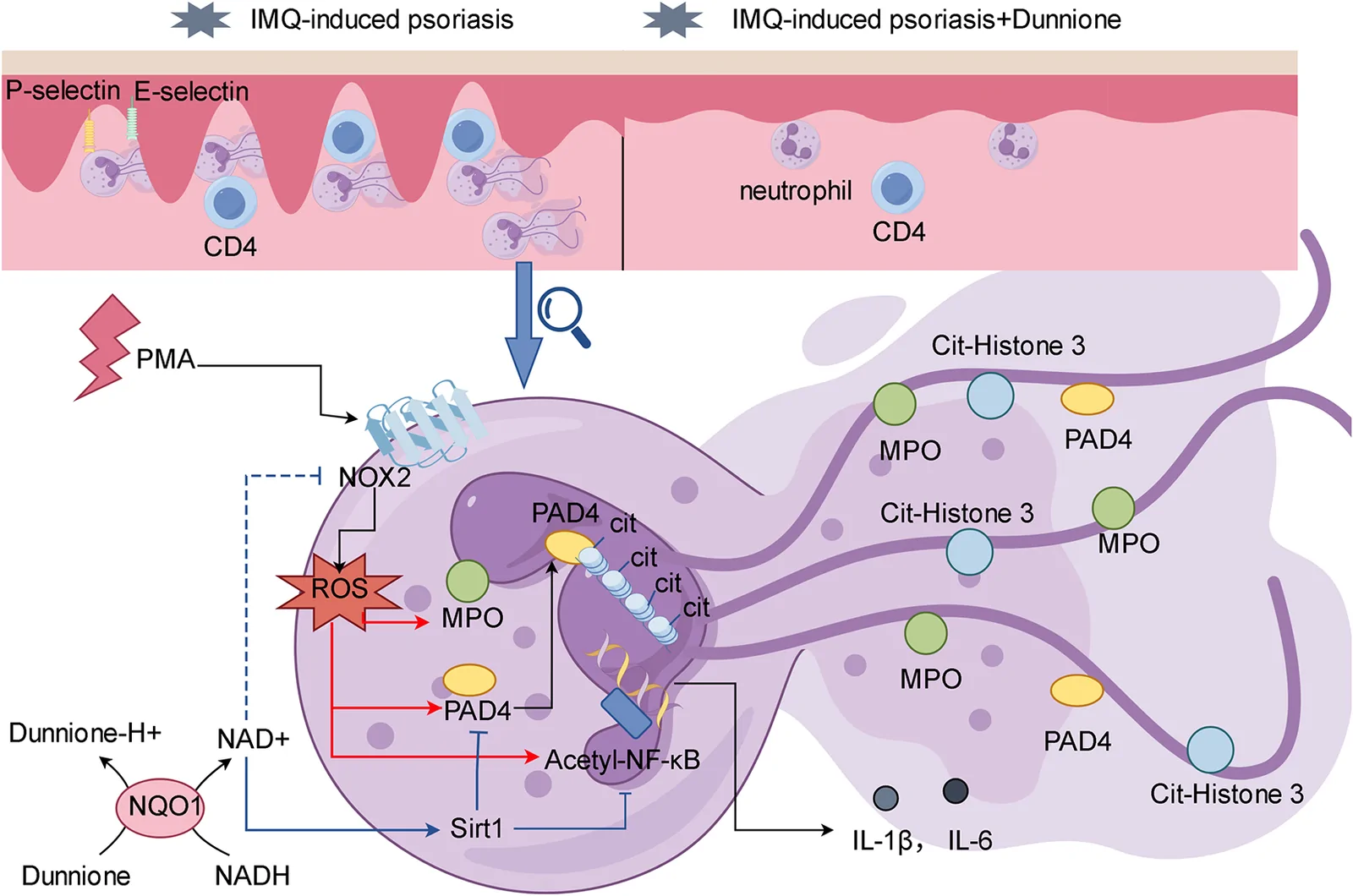
Proposed model of NQO1-dependent redox modulation and SIRT1-associated signaling in NET-mediated inflammation. Dunnione functions as a substrate of NAD(P)H: quinone oxidoreductase 1 (NQO1), promotes NQO1-dependent redox cycling associated with an increased intracellular NAD^+^/NADH ratio. Restoration of redox balance is accompanied by increased SIRT1 activity, reduced NOX2-associated ROS production, decreased PAD4/H3cit responses, and limitation of excessive NET formation. Attenuation of NET release is further associated with reduced NF-κB acetylation and inflammatory cytokine expression. This schematic illustration summarizes a proposed model supported by the present data; the causal necessity of SIRT1 and the *in vivo* requirement for NQO1 remain to be directly established. MPO, myeloperoxidase; NET, neutrophil extracellular trap.

## 5 Conclusion

In conclusion, this study highlights NAD^+^-dependent redox homeostasis as a regulator of NET-associated inflammatory amplification. Our findings indicate that dunnione suppresses NET formation and inflammatory responses through an NQO1-dependent cellular response, accompanied by restoration of the NAD^+^/NADH ratio and SIRT1 activity and by attenuation of ROS-associated signaling, PAD4/H3cit responses, and NF-κB acetylation. These coordinated changes support, but do not definitively prove, a causal role for SIRT1 in dunnione-mediated NET suppression.

Establishing dunnione as a modulator of NAD^+^ redox balance emphasizes the potential value of targeting upstream redox metabolism rather than individual downstream inflammatory mediators. Although psoriasis was used as an experimental model, the NAD^+^-centered redox framework described here may be broadly relevant to inflammatory conditions characterized by dysregulated NET formation. Collectively, these findings provide a rationale for the further exploration of NQO1-dependent redox modulation and NAD^+^-associated signaling as therapeutic targets for chronic inflammatory diseases, while emphasizing that SIRT1-specific manipulation in neutrophils would be required to establish its necessity for dunnione-induced NET suppression.

## Data Availability

The raw data supporting the conclusions of this article will be made available by the authors, without undue reservation.
