# Supplementary material for: Metabolic control of NETosis: dunnione suppresses inflammatory responses through NQO1–dependent redox modulation
Source: Front Immunol. 2026 Oct 22;17:1973758. doi: 10.3389/fimmu.2026.1973758 (PMC13760569; doi:10.3389/fimmu.2026.1973758)
Supplement: Supplementary file 1 [file Supplementaryfile1.docx]

**Supplementary data**

| **Gene names** |  | **Primer sequences** |
| --- | --- | --- |
| IL-1β | primers F | 5’-GCA ACT GTT CCT GAA CTC AAC T-3’, |
|  | primers R | 5’-ATC TTT TGG GGT CCG TCA ACT-3’ |
| IL-6 | primers F | 5’-TAG TCC TTC CTA CCC CAA TTT CC-3’ |
|  | primers R | 5’-TTG GTC CTT AGC CAC TCC TTC-3’ |
| β-actin | primers F | 5’-CAC TGTCGA GTC GCG TCC-3’ |
|  | primers R | 5’-CGC AGC GAT ATC GTC ATC CA-3’ |

**Supplementary Table S1.** Primer sequences for cytokine detection

**Supplementary Figure S1.** Major hematological parameters in IMQ-induced psoriasis-like mice.

Peripheral blood cell counts, including total leukocytes, monocytes, lymphocytes, and neutrophils, were analyzed in control, IMQ-treated, and dunnione-treated mice. Data are presented as mean ± SD (n = 6 per group). Statistical analysis was performed using one-way ANOVA.

**Supplementary Figure S2.** Cytokine profiling and exploratory pathway enrichment analysis in IMQ-induced psoriasis-like mice. (A) Proteome Profiler Mouse XL Cytokine Array analysis of cytokines detected in mouse plasma. Red boxes indicate cytokines with differential signal intensities among the indicated groups. (B) Heatmap showing relative signal intensities of the profiled cytokines. (C) Gene Ontology (GO) and Kyoto Encyclopedia of Genes and Genomes (KEGG) pathway enrichment analyses based on the cytokine profiling results. (D) Heatmap visualization and the pathway enriched analysis. The cytokine-array experiment was performed using two biological replicates per group (n = 2). Data are presented as mean ± SD (n = 2 per group).
